# Improved Proteinuria May Attenuate the Risk of Atrial Fibrillation: A Nationwide Population-Based Cohort Study

**DOI:** 10.3390/jcm13164648

**Published:** 2024-08-08

**Authors:** Yoonkyung Chang, Min Kyoung Kang, Tae-Jin Song

**Affiliations:** 1 Department of Neurology, Mokdong Hospital, Ewha Womans University College of Medicine, Seoul 07985, Republic of Korea; ykchang@ewha.ac.kr; 2 Department of Neurology, Seoul Hospital, Ewha Womans University College of Medicine, Seoul 07804, Republic of Korea; yen101@ewha.ac.kr

**Keywords:** atrial fibrillation, proteinuria, kidney disease, nationwide cohort, incidence

## Abstract

**Background/Objectives**: Proteinuria is documented as a risk factor for atrial fibrillation (AF) and can manifest in either reversible or continued forms. Our objective was to examine the relationship between the change in status for proteinuria and the risk of AF in a longitudinal cohort study on the general population nationwide. **Methods**: We included participants (*n* = 1,708,103) who underwent repetitive health examinations. The presence of proteinuria was determined by dipstick urinalysis results. The outcome was the occurrence of AF (International Classification of Diseases-10 code: I48). **Results**: All included participants, 1,666,111 (97.5%), 17,659 (1.0%), 19,696 (1.2%), and 4637 (0.3%), were categorized into groups of proteinuria-free, improved, progressed, and persistent, respectively. During a median follow-up of 14.5 years, 41,190 (2.4%) cases of AF occurred. In the multivariable analysis, the risk of AF was increased as the initial severity was more severe in the proteinuria-improved and proteinuria-persistent groups (*p* for trend < 0.001). In a further pairwise comparison, the proteinuria-improved group had a relatively lower risk of AF compared to the proteinuria-persistent group (HR: 0.751, 95% CI: 0.652–0.865, *p* < 0.001). **Conclusions**: Our study showed that the risk of AF can change according to alterations in proteinuria status. Notably, recovering from proteinuria can also be considered a modifiable risk factor for AF.

## 1. Introduction

Atrial fibrillation (AF) is associated with a considerable burden of morbidity, including systemic thromboembolism [1,2,3]. AF represents a major and critical risk factor for mortality and stroke, which has been demonstrated [4]. The disease burden of AF continues to increase due to the worldwide trend of population aging and increases in cardiovascular and cerebrovascular diseases [5,6,7]. Therefore, it is important to find both associated factors and risk factors related to AF. Currently, hypertension, cardiomyopathy, smoking, and alcohol have been shown to be associated factors, although additional research, especially the investigation of modifiable risk factors for AF, is urgently needed [8].

Proteinuria is acknowledged as a risk factor for cardiovascular disease and mortality [9,10]. Additionally, proteinuria has been identified as a significant factor that contributes to the occurrence of strokes and coronary heart diseases, irrespective of other cardiovascular risk factors [11]. Moreover, proteinuria has been associated with an elevated risk of hypertension, diabetes, and heart failure in the future [12]. Furthermore, proteinuria is a significant factor in the development of new-onset AF [13,14]. Meanwhile, proteinuria can develop over time, while recovery can occur through associated risk correction or treatment. However, there has not currently been a study with a large enough sample size to determine whether the risk of AF increases according to the improvement or persistence of proteinuria.

We hypothesized that the occurrence risk of AF would be different depending on the recovery from or the persistence of proteinuria. Our objective was to investigate the relationship between shifts in proteinuria status and the risk of AF in a nationwide longitudinal cohort study.

## 2. Materials and Methods

The Korean National Health Insurance System (NHIS) provides thorough coverage of patient characteristics, including demographics, socioeconomic status, medical diagnosis database, and treatment records. It also offers a nationwide health examination database and a database of medical care institutions [15]. It is recommended that individuals with NHIS coverage participate in standardized health examinations [16]. We included participants from the National Health Insurance System–National Health Screening Cohort (NHIS-HEALS). The NHIS-HEALS cohort consists of individuals who have participated in medical health examinations. Their demographics, such as weight, height, smoking habits, alcohol consumption, exercise levels, household income, and comorbidities were obtained. Our study received approval from the Institutional Review Board at Ewha Womans University Seoul Hospital. (Institutional Review Board approval number: SEUMC 2022-02-018).

**Figure 1:**
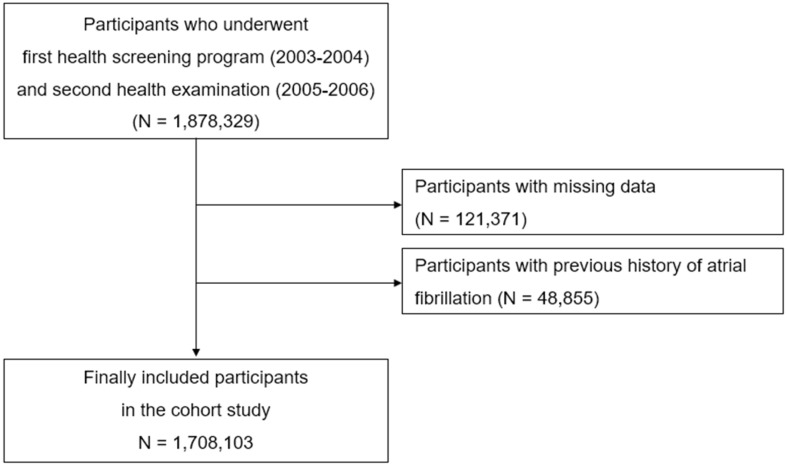
Flowchart depicting the selection process for the study participants.

Our study included participants who undertook repetitive health examinations during both 2003–2004 and 2005–2006 (*n* = 1,878,329). Among these participants, those lacking data for the variables of concern (*n* = 121,371) and those with a previous history of atrial fibrillation (*n* = 48,855) were excluded. Consequently, a total of 1,708,103 participants were involved in the study (Figure 1). In the health screening program, a dipstick urinalysis was conducted after an overnight fasting period to identify the presence or absence of proteinuria. The test results were analyzed using a color scale, which assigned the proteinuria severity as ‘negative’, ‘1 +’, ‘2 +’, ‘3 +’, or ‘4 +’. In this study, we categorized the proteinuria dipstick results into two groups: ‘no proteinuria (-)’ and ‘overt proteinuria (≥1+)’. Afterward, the participants of the study were categorized into four groups: (1) proteinuria-free (0 → 0), (2) proteinuria-improved (1+ → 0, 2+ → ≤1+ (0 or 1+), 3+ ~ 4+ → ≤2+ (0, 1+ or 2+)), (3) proteinuria-progressed (0 → ≥1+, 1+ → ≥2+, 2+ → ≥3+), and (4) proteinuria-persistent (1+ → 1+, 2+ → 2+, 3+ ~ 4+ → 3+ ~ 4+). The day of the second health examination was used as the index date. The primary outcome was the occurrence of AF, as identified by the International Classification of Diseases (ICD)-10 code: I48, with at least two claims per year. The use of codes to classify AF has been validated and employed in previous study [17]. To ensure the accuracy of the AF diagnosis, electrocardiograms were reviewed, and a positive predictive value for AF diagnosis was exhibited as 94.1% [17]. At the index date, the following factors were gathered as covariate: age, sex, body mass index, and household income. Self-administered questionnaires were utilized during health examinations to gather data on smoking, alcohol, and exercise participation. The definitions of the comorbidities are described in the Supplementary Methods [18,19,20,21,22,23].

The baseline characteristics of the groups were compared using appropriate statistical tests: the Chi-square test was employed for categorical variables, while the analysis of variance was employed for continuous variables. The association between the proteinuria status changes and the occurrence of AF was assessed using Kaplan–Meier survival curves with the log-rank test. Cox proportional hazard regression was employed to determine the hazard ratio (HR). In the multivariable Cox regression analysis, different sets of covariates were used for adjustment. Model 1 included age, sex, body mass index, household income, smoking status, alcohol, exercise, and comorbidities, and model 2 further adjusted the Charlson Comorbidity Index. The results of the Cox regression analysis were reported as HR and 95% confidence interval (CI). The assumption of the proportionality of hazards was assessed using Schoenfeld’s residuals, and there was no violation of this assumption detected. The subgroup analysis involved pairwise comparisons to evaluate the altered risk of AF. To minimize the potential impact of reverse causality, a landmark analysis was conducted, with participants with AF within one year of the index date excluded. All statistical analyses were performed using Statistical Analysis System software (SAS version 9.2, SAS Institute, Cary, NC, USA). Statistical significance was defined as a *p*-value < 0.05.

## 3. Results

All included participants were categorized into proteinuria-free, proteinuria-improved, proteinuria-progressed, and proteinuria-persistent groups, which contained 1,666,111 (97.5%), 18,759 (1.10%), 20,936 (1.23%), and 2297 (0.13%) patients, respectively. The median interval between health examinations was found to be 21.5 months (interquartile range, 11.1–25.5 months). Table 1 outlines the baseline characteristics associated with alterations in proteinuria status. The mean age of all participants was 44.0 ± 12.09 years, and 69.0% were male. In the post-hoc analysis, the age and body mass index of the proteinuria-persistent group was highest (*p* < 0.001 for compared to other groups). The group with persistent proteinuria had a significantly higher proportion of male participants compared to the other groups. The group with improved proteinuria exhibited a higher proportion of female participants compared to the other groups. Furthermore, the proteinuria-improved group demonstrated a lower prevalence of current smokers compared to the other groups. Considering the comorbidities, hypertension, diabetes mellitus, dyslipidemia, renal disease, cancer, and a Charlson Comorbidity Index ≥ 2 were lowest in the proteinuria-free group and highest in the proteinuria-persistent group (Table 1).

**Figure 2:**
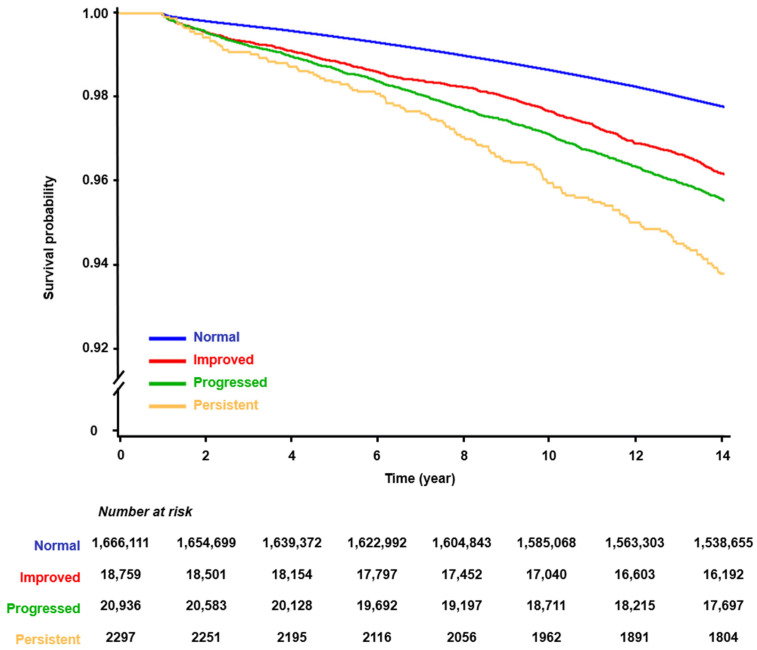
Kaplan–Meier survival curves illustrating the relationship between changes in proteinuria status and atrial fibrillation occurrence.

**Figure 3:**
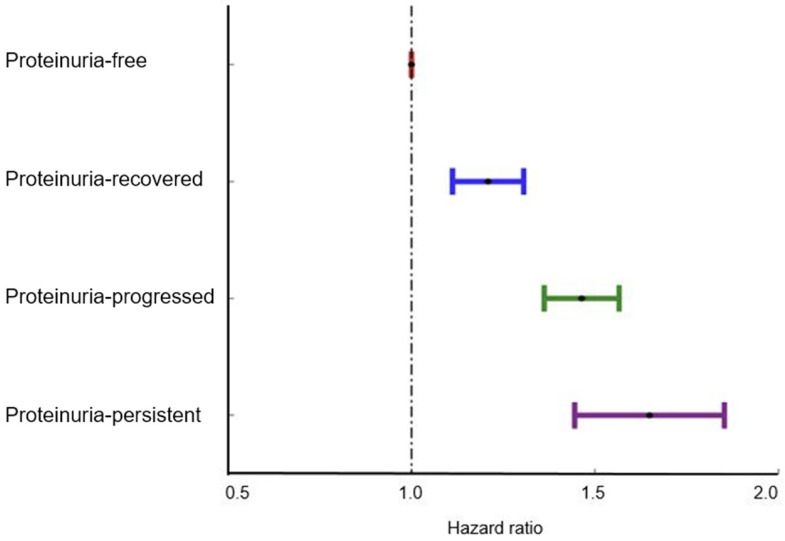
Hazard ratio of atrial fibrillation according to the changes in proteinuria status. The hazard ratio was calculated from the multivariable Cox model adjusted by sex, age, body mass index, household income levels, smoking, alcohol consumption, regular physical activity, hypertension, diabetes mellitus, dyslipidemia, cancer, renal disease, and the Charlson comorbidity index.

**Figure 4:**
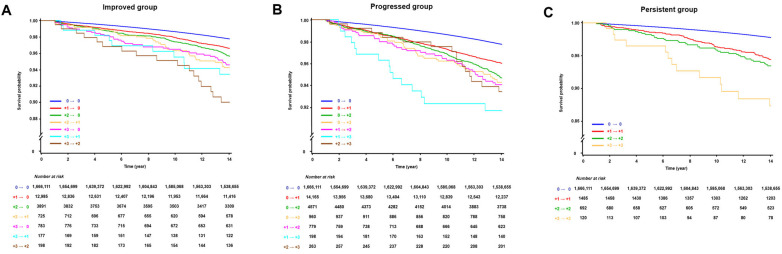
Kaplan–Meier survival curves illustrating the relationship between the changes in proteinuria status and atrial fibrillation occurrence within the improved group (**A**), the progressed group (**B**), and the persistent group (**C**).

During a median follow-up of 14.5 years with an interquartile range of 14.2 to 15.1 years, a total of 41,190 (2.4%) AF cases occurred. Figure 2 presents the Kaplan–Meier survival curves depicting the incidence of AF based on changes in proteinuria status. The participants demonstrated an altered risk of AF depending on their proteinuria status. During the follow-up period, the proteinuria-persistent group exhibited the highest risk of AF, followed by the proteinuria-progressed group, the proteinuria-improved group, and the proteinuria-free group (Figure 2 and Figure 3). In the multivariate analysis (model 2), the risk of AF was increased as the initial severity was more severe in the proteinuria-improved and proteinuria-persistent groups (*p* for trend < 0.001 in each group). Moreover, in the progressed group, the risk of AF was increased according to the severity of proteinuria at the second check (*p* for trend < 0.001) (Table 2, Figure 4). No significant difference in risk was found in the “+1 → 0” group of the proteinuria-improved arm compared to the proteinuria-free arm, which had a higher chance of false positives.

In a further pairwise comparison, there was a relatively lower risk of AF in the proteinuria-improved group than in the proteinuria-persistent group (HR: 0.804, 95% CI: 0.673–0.974, *p* = 0.019). The proteinuria-progressed group demonstrated a higher increased risk of AF compared to the proteinuria-free (HR: 1.474, 95% CI: 1.383–1.575, *p* < 0.001) (Table 3).

Considering the significance of proteinuria, the risk of AF incidents increased according to the severity of the proteinuria, irrespective of the timing of the health examination (Supplementary Table S1) and the presence of renal diseases (Supplementary Table S2).

In the sensitivity analysis, the risk of AF occurring in the proteinuria-improved group (HR: 1.198, 95% CI: 1.109–1.293, *p* < 0.001), proteinuria-progressed group (HR: 1.456, 95% CI: 1.362–1.558, *p* < 0.001), and proteinuria-persistent group (HR: 1.656, 95% CI: 1.476–1.858, *p* < 0.001) was higher than in the proteinuria-free group in multivariable model 3 (Supplementary Table S3). In the landmark analysis, the association between proteinuria status and AF risk was consistently noted (Supplementary Table S4 and Figure S1). Moreover, the pairwise comparison results consistently showed that the proteinuria-improved group had a relatively lower risk of AF compared to the proteinuria-persistent group (HR: 0.783, 95% CI: 0.652–0.912, *p* < 0.001) (Supplementary Table S5).

## 4. Discussion

The key findings in our study indicate that the risk of AF differs based on the change in the proteinuria status. Specifically, we found that the risk of AF decreased following the recovery from proteinuria.

Recent research has provided evidence of a link between proteinuria and a higher likelihood of developing AF. The existing evidence from population-based studies indicates that proteinuria is associated with an elevated risk of AF, irrespective of the concomitant presence of hypertension or diabetes mellitus [24,25]. Another study found that patients with proteinuria also had a higher risk of cardiovascular risk factors, including hypertension, diabetes, obesity, and coronary artery disease. Interestingly, after adjusting for these risk factors, the association between proteinuria and AF remains significant [25]. However, one point that cannot be ignored is that the parameters of these cohorts can change over time. An additional and new finding in our study was that there was a higher risk of AF when proteinuria was persistent for at least 2 years, meaning that the longer the duration of the proteinuria, the higher the risk of AF.

In our study, an improvement in proteinuria led to a significant decrease in the AF risk. There were also several factors that contributed to the modifiable risk factors of AF. Addressing these modifiable risk factors is crucial as part of a comprehensive strategy for the prevention and management of AF. These modifiable risk factors included hypertension, diabetes, obesity, sleep apnea, alcohol consumption, smoking, poor oral hygiene, and physical inactivity [8,26,27]. Moreover, as mentioned previously, while the association between proteinuria and the occurrence of AF is widely recognized, there remain limited studies investigating whether the risk of AF decreases with an improvement in proteinuria. The results of our study further support this, suggesting that proteinuria may also be a modifiable risk factor for AF.

Although the precise mechanism by which persistent or improved proteinuria may influence the AF risk remains unclear, a number of hypotheses can be put forth. It has been demonstrated that proteinuria can give rise to structural alterations in the heart, with the potential for development of left ventricular hypertrophy [28]. Left ventricular hypertrophy (LVH) is a pathological condition marked by the thickening of the cardiac muscle walls of the left ventricle. These structural changes can increase the risk of AF by altering the electrical properties of the heart and promoting the development of abnormal electrical impulses that can trigger AF [29]. Moreover, a reduction in proteinuria may be indicative of enhanced renal function and a decrease in the underlying inflammatory processes that can cause the onset and progression of AF [30,31]. Additionally, proteinuria-associated comorbidities can also increase the risk of AF through several mechanisms, such as endothelial dysfunction, oxidative stress, and systemic inflammation [32]. The link between proteinuria and an elevated risk of developing AF appears to be complex and multifactorial in nature. It is likely that both structural alterations in the heart and the presence of risk factors of AF contribute to this relationship.

Our study has several limitations, whereby, although our study is a longitudinal design, it is not possible to prove the causal relationship in this retrospective design. As the dataset comprised an Asian population, it is challenging to generalize the findings to other populations. As this study utilized standardized health examination data, and we confirmed proteinuria through a single dipstick test, distinguishing whether the transition in proteinuria status resulted from quantitative changes or merely discordance between the two examinations poses a challenge. Moreover, the results of dipstick proteinuria could be influenced by the pH of the urine and other various factors. Since our dataset is a claim dataset, it is difficult to determine whether proteinuria is a false-positive. Furthermore, it is not feasible to adjust for the frequency of all instances of general medical service utilization between screening periods. Although we have demonstrated a dose-dependent relationship to exclude bias as much as possible, cases with positive proteinuria on the first and negative proteinuria on the second measurements are probably false positives or transient proteinuria due to exercise, fever, stress, or food. Lastly, both proteinuria and AF are significantly associated with systemic inflammation, but our claims dataset does not provide information on inflammatory biomarkers.

## 5. Conclusions

In conclusion, our study showed that the risk of AF can change alongside alterations in proteinuria status. Thus, an improvement in proteinuria status can also be considered an amendable risk factor for AF.

## Figures and Tables

**Table 1:** Baseline characteristics of the study participants according to the change in proteinuria.

Variable	Total	Groups According to the Change in Proteinuria				
		Proteinuria-Free	Proteinuria-Improved	Proteinuria-Progressed	Proteinuria-Persistent	*p*-Value
Number of participants (%)	1,708,103	1,666,111 (97.54)	18,759 (1.10)	20,936 (1.23)	2297 (0.13)	
Age, years	44.0 ± 12.09	43.92 ± 12.06	47.01 ± 13.19	47.01 ± 13.05	49.43 ± 12.44	<0.001
Sex						<0.001
Male	1,178,673 (69.00)	1,150,995 (69.08)	11,930 (63.6)	13,943 (66.60)	1816 (78.48)	
Female	529,430 (31.00)	515,116 (30.92)	6829 (36.4)	6993 (33.40)	498 (21.52)	
Body mass index (kg/m^2^)	23.63 ± 3.03	23.61 ± 3.02	24.18 ± 3.33	24.25 ± 3.45	24.89 ± 3.34	<0.001
Household income						<0.001
First quartile, lowest	255,667 (14.97)	248,768 (14.93)	3261 (17.38)	3342 (15.96)	297 (12.83)	
Second quartile	633,817 (37.11)	619,240 (37.17)	6613 (35.25)	7288 (34.81)	680 (29.39)	
Third quartile	564,014 (33.02)	550,552 (33.04)	5891 (31.4)	6763 (32.3)	815 (35.22)	
Fourth quartile, highest	254,605 (14.91)	247,551 (14.86)	2994 (15.96)	3543 (16.92)	522 (22.56)	
Smoking status						<0.001
Never	984,134 (57.62)	958,655 (57.54)	11,620 (61.94)	12,625 (60.3)	1246 (53.85)	
Former	212,855 (12.46)	207,726 (12.47)	2216 (11.81)	2546 (12.16)	370 (15.99)	
Current	511,114 (29.92)	499,730 (29.99)	4923 (26.24)	5765 (27.54)	698 (30.16)	
Alcohol consumption (days/week)						<0.001
<3	1,143,895 (66.97)	1,115,187 (66.93)	12,972 (69.15)	14,239 (68.01)	1512 (65.34)	
≥3	564,208 (33.03)	550,924 (33.07)	5787 (30.85)	6697 (31.99)	802 (34.66)	
Regular exercise (days/week)						<0.001
<3	1,377,901 (80.67)	1,344,948 (80.72)	14,564 (77.64)	16,603 (79.3)	1800 (77.79)	
≥3	330,202 (19.33)	321,163 (19.28)	4195 (22.36)	4333 (20.7)	514 (22.21)	
Comorbidities						
Hypertension	773,343 (45.27)	748,007 (44.9)	11,062 (58.97)	12,529 (59.84)	1762 (76.15)	<0.001
Diabetes mellitus	241,826 (14.16)	230,155 (13.81)	4991 (26.61)	5775 (27.58)	915 (39.54)	<0.001
Dyslipidemia	423,808 (24.81)	408,103 (24.49)	6932 (36.95)	7674 (36.65)	1113 (48.1)	<0.001
Cancer	31,617 (1.85)	30,441 (1.83)	536 (2.86)	556 (2.66)	85 (3.67)	<0.001
Renal disease	17,424 (1.02)	15,207 (0.91)	1002 (5.34)	934 (4.46)	283 (12.23)	<0.001
Charlson Comorbidity Index						
0	677,173 (39.64)	663,858 (39.84)	5941 (31.67)	6762 (32.3)	612 (26.45)	<0.001
1	692,943 (40.57)	677,737 (40.68)	6794 (36.22)	7706 (36.81)	710 (30.68)	<0.001
≥2	337,987 (19.79)	324,516 (19.48)	6024 (32.11)	6468 (30.89)	992 (42.87)	<0.001

Data are expressed as mean ± standard deviation or *n* (%).

**Table 2:** Multivariable Cox analysis for the occurrence of atrial fibrillation based on the change in proteinuria.

	Number of Events	Event Rate (%)	Incidence Rate (Per 1000 Person Years)	Unadjusted		Multivariable Adjusted (1)		Multivariable Adjusted (2)	
				HR (95% CI)	*p* Value	HR (95% CI)	*p* Value	HR (95% CI)	*p* Value
Atrial Fibrillation	40,654	2.38	1.68						
Proteinuria-Free: 0 → 0	38,857	2.33	1.64	1 (Ref)		1 (Ref)		1 (Ref)	
Proteinuria-Improved									
+1 → 0	438	3.37	2.43	1.48 (1.35, 1.63)	<0.001	1.11 (1.01, 1.22)	0.035	1.10 (1.00, 1.21)	0.053
+2 → 0	172	4.42	3.23	1.98 (1.70, 2.29)	<0.001	1.43 (1.23, 1.66)	<0.001	1.40 (1.21, 1.63)	<0.001
+2 → +1	41	5.66	4.25	2.63 (1.93, 3.57)	<0.001	1.59 (1.17, 2.16)	0.003	1.56 (1.15, 2.12)	0.004
+3~4 → 0	43	5.49	4.15	2.55 (1.89, 3.44)	<0.001	1.83 (1.36, 2.47)	<0.001	1.80 (1.33, 2.42)	<0.001
+3~4 → +1	10	5.65	4.59	2.87 (1.54, 5.33)	0.009	1.54 (1.03, 2.04)	0.017	1.53 (1.02, 2.04)	0.0191
+3~4 → +2	18	9.09	7.34	4.59 (2.89, 7.29)	<0.001	2.53 (1.59, 4.02)	<0.001	2.48 (1.56, 3.93)	0.001
Proteinuria-Progressed									
0 → +1	566	4	2.91	1.78 (1.63, 1.93)	<0.001	1.36 (1.26, 1.48)	<0.001	1.36 (1.25, 1.47)	<0.001
0 → +2	238	5.21	3.87	2.38 (2.09, 2.70)	<0.001	1.64 (1.44, 1.86)	<0.001	1.62 (1.43, 1.84)	<0.001
0 → +3~4	55	5.73	4.35	2.69 (2.06, 3.50)	<0.001	2.07 (1.59, 2.69)	<0.001	2.06 (1.58, 2.68)	<0.001
+1 → +2	46	5.91	4.46	2.74 (2.06, 3.66)	<0.001	1.45 (1.09, 1.94)	0.011	1.44 (1.08, 1.93)	0.013
+1 → +3~4	17	8.59	6.93	4.33 (2.69, 6.96)	<0.001	2.37 (1.48, 3.82)	0.004	2.32 (1.44, 3.74)	0.005
+2 → +3~4	16	6.08	4.71	2.92 (1.79, 4.77)	<0.001	1.80 (1.10, 2.93)	0.019	1.75 (1.07, 2.86)	0.024
Proteinuria-Persistent									
+1 → +1	81	5.45	4.05	2.49 (2.01, 3.1)	<0.001	1.45 (1.17, 1.8)	0.008	1.43 (1.15, 1.78)	0.001
+2 → +2	43	6.21	4.78	2.96 (2.20, 3.99)	<0.001	1.65 (1.23, 2.23)	0.001	1.64 (1.22, 2.22)	0.001
+3~4 → +3~4	13	10.83	9.18	5.79 (3.36, 9.96)	<0.001	3.53 (2.05, 6.08)	<0.001	3.47 (2.01, 5.97)	<0.001

Multivariable model (1) was adjusted for age, sex, body mass index, household income, smoking status, alcohol consumption, regular exercise, and comorbidities. Multivariable model (2) was adjusted for the variables listed above as well as the Charlson Comorbidity Index. HR, hazard ratio, CI, confidence interval.

**Table 3:** Pairwise comparisons of the association between the change in proteinuria status and the occurrence of atrial fibrillation.

Status of Proteinuria	Multivariable Adjusted	
	HR (95% CI)	*p* Value
Improved vs. Free (reference)	1.233 (1.142, 1.333)	<0.001
Progressed vs. Free (reference)	1.474 (1.383, 1.575)	<0.001
Persistent vs. Free (reference)	1.595 (1.345, 1.886)	<0.001
Improved vs. Persistent (reference)	0.804 (0.673, 0.974)	0.019
Progressed vs. Persistent (reference)	0.947 (0.786, 1.126)	0.466
Improved vs. Progressed (reference)	0.847 (0.767, 0.938)	0.005

The multivariable model was adjusted for age, sex, body mass index, household income, smoking status, alcohol consumption, regular exercise, comorbidities, and the Charlson Comorbidity Index. HR, hazard ratio, CI, confidence interval.

## Data Availability

The data that support the findings of this study are available from NHIS-HEALS; however, restrictions apply to the availability of these data, which were used under license for the current study and, hence, are not publicly available. However, data are available from the authors upon reasonable request and with permission from the National Health Insurance System.

## Supplementary Materials

The following supporting information can be downloaded at: https://www.mdpi.com/article/10.3390/jcm13164648/s1, Supplementary Methods, Figure S1: Kaplan–Meier survival curves illustrating the relationship between changes in the proteinuria status and atrial fibrillation occurrence after excluding those who developed an atrial fibrillation event in the first year; Table S1: Relationship between the severity of proteinuria and atrial fibrillation incidence according to the timing of health examination; Table S2: Relationship between the severity of proteinuria and atrial fibrillation incidence according to the presence of renal disease; Table S3: Relationship between the proteinuria status and atrial fibrillation incidence; Table S4: Relationship between the proteinuria status and atrial fibrillation incidence after excluding those who developed an atrial fibrillation event in the first year; Table S5: Comparison of incident atrial fibrillation according to the proteinuria status after excluding those who developed an atrial fibrillation event in the first year [18,33,34,35,36,37,38].
