# Supplementary material for: Improved Proteinuria May Attenuate the Risk of Atrial Fibrillation: A Nationwide Population-Based Cohort Study
Source: J Clin Med. 2024 Aug 8;13(16):4648. doi: 10.3390/jcm13164648 (PMC11355726; doi:10.3390/jcm13164648)
Supplement: Supplementary file 1 [file jcm-13-04648-s001.zip › jcm-3070884-supplementary.pdf]

Title: Recovered Proteinuria May Attenuate the Risk of Atrial fibrillation: A nationwide population-based cohort study

*[1]Younkyung Chang, MD, PhD, *[2]Min Kyoung Kang, MD, PhD, [2]Tae-Jin Song MD, PhD

[1]Department of Neurology, Mokdong Hospital, Ewha Womans University College of Medicine, Seoul, Republic of Korea

[2]Department of Neurology, Seoul Hospital, Ewha Womans University College of Medicine, Seoul, Republic of Korea

*: equally contributed.

Correspondence:

Tae-Jin Song, MD, PhD.

Department of Neurology, Seoul Hospital, Ewha Womans University College of Medicine, 260, Gonghang-daero, Gangseo-gu, Seoul, 07804, Korea

Tel: +82-2-6986-2010, Fax: +82-2-6986-2111; E-mail: knstar@ewha.ac.kr

ORCID: 0000-0002-9937-762X

Supplementary Methods

Definition of covariates

Smoking history (never, former, and current)

Definition and measurement of variables like usual smoking habits were obtained by questionnaire in the health examination program. Smoking status was used to categorize participants into three groups: none, former smoker, and current smoker. Current smoker was classified according to the WHO definition as a person who has smoked more than five packs (100 cigarettes) in a lifetime and smoked daily or occasionally for the last 28 days. Former smoker was defined as a person who had smoked more than 100 cigarettes in a lifetime and had not smoked in the last 28 days [1]

Alcohol consumption (none, moderate, and heavy)

Definition and measurement of variables like usual alcohol consumption were obtained by questionnaire in the health examination program. The frequency of alcohol consumption was dichotomized into <3 days per week or ≥3 days per week, regardless of the amount of the drink. [2]

Regular physical activity

Definition and measurement of variables like usual regular physical activity were obtained by questionnaire in the health examination program. The frequency of regular physical activity was dichotomized into <3 days per week or ≥3 days per week, regardless of the intensity or duration of the exercise. [3]

Comorbidities

Hypertension

Hypertension was defined as using at least one claim of ICD-10 code(I10-15) with the prescription of an anti-hypertensive agent, claims of ICD-10 code(I10-15) more than two times, a systolic blood pressure of ≥140 mmHg and a diastolic blood pressure of ≥90 mmHg or positive checking in self-report questionnaire on hypertension in the health examination program. [4]

Diabetes mellitus

Diabetes mellitus was defined as satisfying one of following criteria: 1) at least one claim of diagnostic codes (ICD-10 E11–14) with the prescription of an antidiabetic agent, 2) two or more

claims of diagnostic codes (ICD-10 E11–14), 3) fasting serum glucose level ≥ 7.0 mmol/L, or 4) self-reported diabetes mellitus in the questionnaire. [4]

Dyslipidemia

Dyslipidemia was defined as using at least one claim of ICD-10 code(E78) with the prescription of an anti-dyslipidemic agent, claims of ICD-10 code(E78) more than two times or total cholesterol level of ≥240 mg/dL. [5]

Cancer

Cancer was defined as having once admission or at least three times outpatient claims of diagnostic code (ICD-10 C00–97) with specific registration code of 'V027' or 'V193–4'. [5]

Renal disease

Renal disease was defined as two or more claims of diagnostic codes (ICD-10 N17-19, I12-13, E082, E102, E112, E132), or estimated glomerular filtration rate less than 60 $mL/min/1.73m^2$. [5]

Estimated glomerular filtration rate (eGFR)

The eGFR was calculated from serum creatinine (SCr) at the time of hospital visit using the Chronic Kidney Disease Epidemiology Collaboration (CKD-EPI) equation: $eGFR=144\times(SCr/0.7)^{-0.329}\times(0.993)^{age}$ (if female and SCr≤0.7 mg/dL), $eGFR=144\times(SCr/0.7)^{-1.209}\times(0.993)^{age}$ (if female and SCr>0.7 mg/dL), $eGFR=144\times(SCr/0.9)^{-0.411}\times(0.993)^{age}$ (if male and SCr≤0.9 mg/dL), and $eGFR=144\times(SCr>0.9)^{-1.209}\times(0.993)^{age}$ (if male and SCr>0.9 mg/dL). [6]

Charlson comorbidity index (0, 1, or ≥2).

The Charlson comorbidity index score was calculated for each subject based on diseases diagnosed before index date and divided into three groups (0, 1, and ≥2 scores). [7]

References

1. Lee, K. H., Lee, C. M., Kwon, H. T. & Oh, S.-W. Relationship between Obesity and Smoking in Korean Men: Data Analyses from the Third and Fourth Korea National Health and Nutrition Examination Surveys (KNHANES). JKSRNT 1, 115-123 (2010). https://doi.org:10.25055/JKSRNT.2010.1.2.115

2. Tu, S. J. et al. Risk Thresholds for Total and Beverage-Specific Alcohol Consumption and Incident Atrial Fibrillation. JACC Clin Electrophysiol 7, 1561-1569 (2021). https://doi.org:10.1016/j.jacep.2021.05.013

3. Son J.H et al. Optimal Frequency Intensity of Physical Activity to Reduce the Risk of Hypertension in the Korean Population. Exerc Sci, 31, 129-140 (2022). https://doi.org/10.15857/ksep.2021.00626

4. Song, T. J., Kim, J. W. & Kim, J. Oral health and changes in lipid profile: A nationwide cohort study. J Clin Periodontol 47, 1437-1445 (2020). https://doi.org:10.1111/jcpe.13373

5. Park, J. H., Chang, Y., Kim, J. W. & Song, T. J. Improved Oral Health Status Is Associated with a Lower Risk of Venous Thromboembolism: A Nationwide Cohort Study. J Pers Med 13 (2022). https://doi.org:10.3390/jpm13010020

6. Tent, H. et al. Performance of MDRD study and CKD-EPI equations for long-term follow-up of nondiabetic patients with chronic kidney disease. Nephrol Dial Transplant 27 Suppl 3, iii89-95 (2012). https://doi.org:10.1093/ndt/gfr235

7. Kim, K.H. Comorbidity Adjustment in Health Insurance Claim Database. Health Policy and Management. Health Policy and Management, 26, 71-78 (2016). https://doi.org/10.4332/KJHPA.2016.26.1.71

Supplementary Table S1. Relationship between severity of proteinuria and atrial fibrillation incidence according to the timing of health examination

Severity of proteinuria	Number of participants	Number of events	Incidence rate (per 1,000 person-years)	Model 1 HR (95% CI)	*P* for trend	Model 2 HR (95% CI)	*P* for trend	Model 3 HR (95% CI)	*P* for trend
				First period (2003 or 2004)					
Negative	1,685,807	40,231	1.68	1 (reference)	< 0.001	1 (reference)	< 0.001	1 (reference)	< 0.001
1+	15,447	594	2.79	1.664 (1.534, 1.804)		1.182 (1.090, 1.282)		1.171 (1.080, 1.270)	
2+	5,571	278	3.70	2.212 (1.966, 2.489)		1.493 (1.326, 1.680)		1.469 (1.305, 1.653)	
3+	1,131	73	5.04	3.034 (2.412, 3.817)		1.943 (1.544, 2.446)		1.892 (1.503, 2.382)	
4+	147	14	7.44	4.499 (2.667, 7.592)		3.005 (1.781, 5.068)		3.106 (1.841, 5.240)	
				Second period (2005-2006)					
Negative	1,683,770	40,022	1.68	1 (reference)	< 0.001	1 (reference)	< 0.001	1 (reference)	< 0.001
1+	16,552	707	3.13	1.872 (1.738, 2.017)		1.378 (1.279, 1.485)		1.369 (1.270, 1.474)	
2+	6,240	356	4.29	2.581 (2.326, 2.865)		1.668 (1.502, 1.852)		1.650 (1.486, 1.832)	
3+	1,289	92	5.54	3.362 (2.741, 4.123)		2.265 (1.846, 2.779)		2.241 (1.826, 2.749)	
4+	252	13	4.05	2.456 (1.426, 4.230)		1.954 (1.134, 3.366)		1.913 (1.110, 3.295)	

Model 1: unadjusted model.

Model 2: sex, age, body mass index, household income levels, smoking, alcohol consumption, regular physical activity, hypertension, diabetes mellitus, dyslipidemia, cancer, and renal disease were adjusted.

Model 3: sex, age, body mass index, household income levels, smoking, alcohol consumption, regular physical activity, hypertension, diabetes mellitus, dyslipidemia, cancer, renal disease and Charlson comorbidity index were adjusted.

HR, hazard ratio.; CI, confidence interval.

Supplementary Table S2. Relationship between severity of proteinuria and atrial fibrillation incidence according to the presence of renal disease

Severity of proteinuria	Number of participants	Number of events	Incidence rate (per 1,000 person-years)	Hazard ratio (95% CI)	*P* for trend
			Presence of renal disease		
Negative	16,021	1,041	4.90	1 (reference)	< 0.001
1+	704	49	5.60	1.197 (0.897-1.5974)	
2+	489	48	8.33	1.703 (1.272-2.280)	
3+	180	21	10.47	2.351 (1.522-3.634)	
4+	30	3	79.00	2.744 (0.881, 8.549)	
			Absence of renal disease		
Negative	1,667,749	38,981	1.65	1 (reference)	< 0.001
1+	15,848	658	3.03	1.375 (1.273, 1.485)	
2+	5,751	308	3.99	1.628 (1.455, 1.822)	
3+	1,109	71	4.86	2.138 (1.694, 2.699)	
4+	222	10	3.48	2.456 (0.989, 3.138)	

Sex, age, body mass index, household income levels, smoking, alcohol consumption, regular physical activity, hypertension, diabetes mellitus, dyslipidemia, cancer and Charlson comorbidity index were adjusted.

CI, confidence interval.

Supplementary Table S3. Relationship between proteinuria status and atrial fibrillation incidence.

Group	Number of participants	Number of events	Incidence rate (per 1,000 person-years)	Model 1 HR (95% CI)	Model 2 HR (95% CI)	Model 3 HR (95% CI)	*P*-value
Proteinuria-free	1,666,111	39,358	1.67	1 (reference)	1 (reference)	1 (reference)	
Proteinuria-improved	17,659	664	2.73	1.640 (1.519, 1.771)	1.211 (1.121, 1.308)	1.198 (1.109, 1.293)	< 0.001
Proteinuria-progressed	19,696	873	3.25	1.961 (1.834, 2.097)	1.467 (1.371, 1.569)	1.456 (1.362, 1.558)	< 0.001
Proteinuria-persistent	4,637	295	4.87	2.961 (2.641, 3.320)	1.679 (1.496, 1.883)	1.656 (1.476, 1.858)	< 0.001

Model 1: unadjusted model.

Model 2: sex, age, body mass index, household income levels, smoking, alcohol consumption, reg-ular physical activity, hypertension, diabetes mellitus, dyslipidemia, cancer, and renal disease were adjusted.

Model 3: sex, age, body mass index, household income levels, smoking, alcohol consumption, reg-ular physical activity, hypertension, diabetes mellitus, dyslipidemia, cancer, renal disease and Charlson comorbidity index were adjusted.

HR, hazard ratio; CI, confidence interval.

Supplementary Table S4. Relationship between proteinuria status and atrial fibrillation incidence after excluding those who developed atrial fibrillation event in the first year

Group	Number of participants	Number of events	Incidence rate (per 1,000 person-years)	Model 1 HR (95% CI)	Model 2 HR (95% CI)	Model 3 HR (95% CI)	*P*-value
Proteinuria-free	1,666,111	38,857	1.64	1 (reference)	1 (reference)	1 (reference)	
Proteinuria-recovered	17,659	653	2.68	1.634 (1.512, 1.765)	1.209 (1.119, 1.306)	1.196 (1.107, 1.292)	< 0.001
Proteinuria-developed	19,696	859	3.20	1.955 (1.827, 2.091)	1.464 (1.368, 1.566)	1.453 (1.358, 1.555)	< 0.001
Proteinuria-chronic	4,637	285	4.69	2.896 (2.578, 3.254)	1.649 (1.467, 1.853)	1.626 (1.447, 1.828)	< 0.001

Model 1: unadjusted model.

Model 2: sex, age, body mass index, household income levels, smoking, alcohol consumption, regular physical activity, hypertension, diabetes mellitus, dyslipidemia, cancer, and renal disease were adjusted.

Model 3: sex, age, body mass index, household income levels, smoking, alcohol consumption, regular physical activity, hypertension, diabetes mellitus, dyslipidemia, cancer, renal disease and Charlson comorbidity index were adjusted.

HR, hazard ratio; CI, confidence interval.

Supplementary Table S5. Comparison of incident atrial fibrillation according to proteinuria status after excluding those who developed atrial fibrillation event in the first year

Group	Model 1 HR (95% CI)	*P*-value
Proteinuria-recovered vs Proteinuria-free (reference)	1.121 (1.106, 1.138)	< 0.001
Proteinuria-developed vs Proteinuria-free (reference)	1.432 (1.362, 1.501)	< 0.001
Proteinuria- recovered vs Proteinuria-chronic (reference)	0.783 (0.625, 0.912)	< 0.001
Proteinuria- developed vs Proteinuria-chronic (reference)	0.889 (0.779, 0.9988)	0.042

Sex, age, body mass index, household income levels, smoking, alcohol consumption, regular physical activity, hypertension, diabetes mellitus, dyslipidemia, cancer and Charlson comorbidity index were adjusted.

HR, hazard ratio; CI, confidence interval.

Supplementary Figure

Supplementary Figure S1. Kaplan-Meier survival curves illustrating the relationship between changes in proteinuria status and atrial fibrillation occurrence after excluding those who developed atrial fibrillation event in the first year.

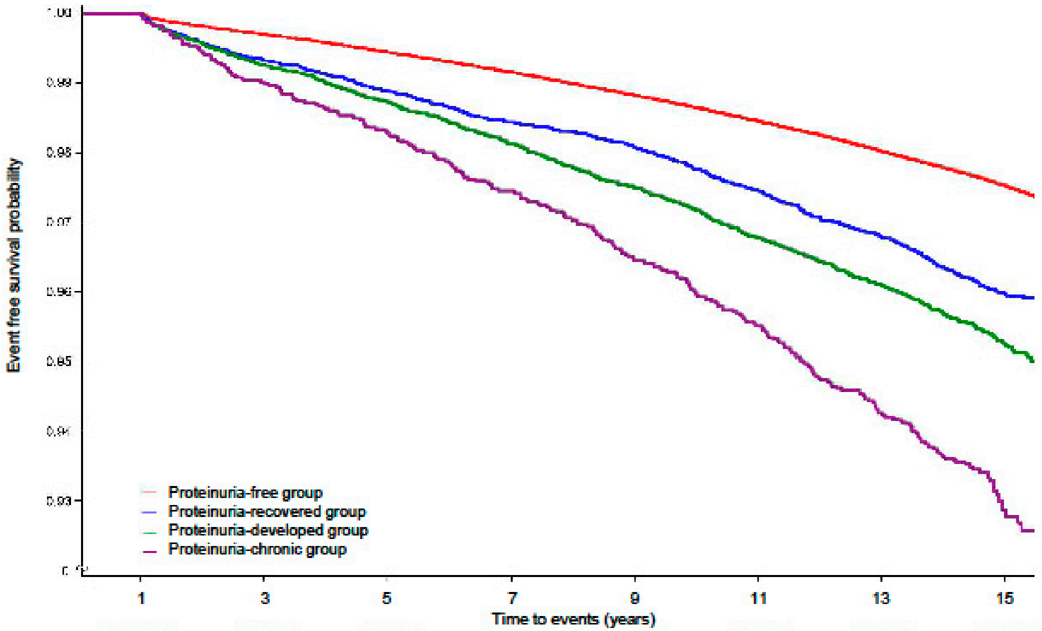

	1	3	5	7	9	11	13	15
Proteinuria-free group	1662971	1647155	1631352	1614202	1595288	1574538	1551250	496841
Proteinuria-recovered group	17586	17272	16956	16636	16323	15951	15561	5618
Proteinuria-developed group	19586	19160	18785	18350	17896	17455	16986	6281
Proteinuria-chronic group	4602	4470	4323	4164	4010	3849	3691	1257
